# Assessing the psychosocial work environment in the health care setting: translation and psychometric testing of the French and Italian Copenhagen Psychosocial Questionnaires (COPSOQ) in a large sample of health professionals in Switzerland

**DOI:** 10.1186/s12913-022-07924-4

**Published:** 2022-05-06

**Authors:** Karin Anne Peter, Christoph Golz, Reto Arthur Bürgin, Matthias Nübling, Christian Voirol, Simeon Joel Zürcher, Sabine Hahn

**Affiliations:** 1 Department of Applied Research & Development in Nursing, Bern University of Applied Sciences, Murtenstrasse 10, 3008 Bern, Switzerland; 2 Freiburg Research Centre for Occupational Sciences, Freiburg, Germany; 3 Haute Ecole Arc Santé, University of Applied Sciences and Arts Western Switzerland, Neuchatel, Switzerland

**Keywords:** Psychosocial risks, Occupational health and safety, Healthcare sector, Validation, Switzerland

## Abstract

**Background:**

Measuring work-related stress in a reliable way is important in the development of appropriate prevention and intervention strategies. Especially in multilingual studies the use of comparable and reliable instruments is crucial. Therefore, the aim of this study was to translate selected scales and single items from the German version of the Copenhagen Psychosocial Questionnaire (COPSOQ) into French and Italian and psychometrically test them in a sample of health professionals.

**Methods:**

This study used cross-sectional data from health professionals at 163 randomised selected health organisations in Switzerland. Selected COPSOQ items/scales were backwards- and forwards- translated and cross-culturally adapted from German to French and Italian. Reliability was assessed with Cronbach alpha and intraclass correlation coefficients, construct validity with confirmatory factor analysis (CFA) and structural equation modelling as well as comparative fit index.

**Results:**

Responses from 12,754 health professionals were included in the analysis. Of the overall 24 scales, 20 in the German version, 19 in the French version and 17 in the Italian version attained sufficient internal consistency with a threshold of 0.7 for Cronbach’s alpha. Predominantly high factor loadings on scale level are reported (> 0.35), as well as good and satisfactory fit values with RMSEA below 0.1, SRMR below 0.08 and CFI above 0.95. For 10 out of 15 scales, the test for factor invariance revealed a significant difference regarding the psychological constructs of the scales across the language versions.

**Conclusions:**

The psychometric properties verify the underlying theoretical model of the COPSOQ questionnaire, which is to some extent comparable across the three language versions. Of the 10 scales with significant factor variance, four showed large differences, implying that revision is needed for better comparability. Potential cultural issues as well as regional differences may have led to the factor variance and the different reliability scores per scale across language versions. One known influencing factor for regional differences is culture, which should be considered in scale development. Moreover, emerging topics such as digitization should be considered in further development of the questionnaire.

**Supplementary Information:**

The online version contains supplementary material available at 10.1186/s12913-022-07924-4.

## Background

Stress at work is becoming an increasingly relevant issue, with one in six European employees reporting chronic health problems [1]. The resulting costs of stress at work are internationally considered a significant financial burden on society (US$ 221′13 million to 187 billion) [2]. In Switzerland, for example, work-related stress accounts for 24% of total health-related production losses due to absenteeism as well as presenteeism, which corresponds to 3.2% of employees’ average monthly earnings [3]. Work-related stress is defined as ‘a pattern of reactions that occurs when workers are presented with demands or pressures (stressors) that are not matched to their knowledge, abilities and skills and which challenge their ability to cope’ [4, 5].

Health professionals in particular are frequently affected by various stressors at work, such as work-private life conflicts, understaffing, long working hours, high quantitative and emotional demands and reward frustration [6–10]. Stress at work potentially leads to lower job satisfaction and commitment to the organization, and is associated with health professionals’ intention to leave their profession prematurely [11–13]. In consequence, work-related stress may exacerbate the issue of workforce shortage of qualified health professionals in several countries [14]. In Switzerland, the healthcare system is also struggling with such a shortage [15].

Assessment tools that capture stressors and consequences of stress at work among health professionals in a reliable and valid way are essential in developing appropriate prevention and intervention strategies. Several studies have been conducted to assess work-related stress and intention to leave among health professionals, such as the European longitudinal Nurses’ Early Exit study [16–18] or the RN4CAST [19] study, using selected scales of the Copenhagen Psychosocial Questionnaire (COPSOQ) to cover relevant topics among health professionals. The COPSOQ developed by Kristensen [20] is one of the most widely used instruments and has been translated into more than 25 languages [21–23]. The COPSOQ is a self-report questionnaire that assesses psychosocial stressors and stress reactions as well as individual health and well-being [5], and has the advantage of a scientifically grounded theoretical background [24]. The COPSOQ is available in a short, middle or long version and is designed for workplace surveys, analytic research and international comparisons [5, 20, 22]. The scales and single items included in the COPSOQ, are used to assess various stressors at work, such as demands (e.g. quantitative demands, sensorial demands), work organisation and content (e.g. influence at work, opportunities for development, meaning of work), social relations and leadership (e.g. predictability of work, role clarity, role conflicts, quality of leadership, social support at work), the person-work interface (e.g. job insecurity) as well as the home-work interface (e.g. work-private life conflict, demarcation). In addition, scales assessing employees’ stress reaction (e.g. behavioural or cognitive stress symptoms) and possible long-term consequences of stress at work (e.g. burnout-symptoms) are included [22].

The COPSOQ has already been used in the healthcare sector, translated and validated in German, French and Italian and tested in previous studies [17, 25–28]. The current version, number 3, of COPSOQ developed by the International COPSOQ Network [29] consists of so-called core items that are mandatory in any national version and further items that can be added. Thus, every national version differs in these further questions. Consequently, since the available translated versions have been adapted to the cultural conditions of the country for which they were designed and differ greatly in terms of topics and item selection, comparable French, Italian and German versions of the questionnaire for multilingual studies are currently lacking. As an outlook for further developments of the questionnaire, the COPSOQ international network strives for international comparability and calls to examine validity across countries [25]. A comparable version in German, French and Italian is especially important for countries with these national languages, such as Switzerland (66% German-speaking, 23% French-speaking, 8% Italian-speaking). In multilingual samples like Switzerland, cultural adaptation is important to understand if the linguistic groups interpret and understand the items in the same way. Therefore, comparable items / scales are essential [30].

This study aims to present selected scales and single items from the German COPSOQ Version translated into French and Italian and to analyse their psychometric properties in a large and heterogeneous sample of health professionals in Switzerland.

## Methods

### Design

This study was conducted in two phases. First, the selected scales and single items from the COPSOQ were translated from German into French/Italian, culturally adapted and tested using ‘cognitive debriefing’ in interviews.

Second, the translated scales and single items were psychometrically validated in a large group of health professionals as part of the **STRAIN** project (work-related **str**ess **a**mong health professionals **in** Switzerland). Briefly, STRAIN is an ongoing cluster randomized controlled trial (ClinicalTrials.gov identifier: NCT03508596) that is based on three measurements: the baseline T^0^, the first measure T^1^ and second measure T^2^. The results presented in this study are based on the cross-sectional data from the STRAIN baseline measurement T^0^ (September 2017 to March 2018) and the first measurement T^1^ (January to May 2019). Since cases with repeated measurements were identified and removed (e.g. if a person filled out the questionnaire at T^0^ and T^1^, the case at T^1^ was removed) the study is based on cross-sectional data only. Further details regarding the STRAIN project are published in Peter, Schols [31].

### Recruitment and study sample

Health organisations were randomly selected from all hospitals, nursing homes, and home care organisations registered by the Swiss Federal Statistical Office in 2016. These included Swiss acute care, rehabilitation and psychiatric hospitals, nursing homes and home care organizations from all language regions of Switzerland. A total of 100 hospitals, 100 nursing homes, and 100 home care organisations were randomly selected from the German, French, and Italian-speaking regions of Switzerland using a web-based randomization approach [32] also ensuring a geographically representative sample for Switzerland. Overly small (average number of beds < 20, < 7 employees) or specialised organisations (e.g. in gynaecology or neonatology) were excluded.

Selected organisations were invited to participate and provided with information about the study. A total of 36 acute care, rehabilitation or psychiatric hospitals (23 German-speaking, 12 French-speaking, 1 Italian-speaking), 86 nursing homes (56 German-speaking, 24 French-speaking, 6 Italian-speaking) and 41 home care organisations (36 German-speaking, 3 French-speaking, 2 Italian-speaking) agreed to take part in the study [31].

### Content and use of the questionnaire

Using the German COPSOQ versions from 2005 and the extended German standard version 2017 ([26]; Nübling et al. 2017 [33]), we selected scales for translation and validation that were in previous studies [34] considered relevant regarding the work environment and demands at work in the healthcare sector. Table 1 shows the seven domains and 29 selected COPSOQ scales that were translated and validated for this study. All questions (i.e. items) for the three languages are available in Supplement A. For all scales used in the questionnaire, consent was obtained from the original author for their use. The COPSOQ versions are not under license. The scales we included from COPSOQ revealed satisfactory-good construct validity, criterion validity, diagnostic power and reliability (Cronbach’s alpha 0.64–0.89) in previous studies [22, 25, 26].

**Table 1:** Domains, scales and number of items per scale in the German, French, and Italian short/modified version of COPSOQ

Domain	Scale (examples)	Number of Items
**Demands at Work**	Quantitative demands (working at a high pace, doing overtime)	3
	Sensorial demands (precision, vision, attention)	5
	Work environment (being exposed to noise, cold, chemicals)	5
	Demands for hiding emotions (hiding feelings)	2
**Work organisation & content**	Opportunities for development (opportunity to develop skills)	3
	Influence at work (degree of influence concerning work)	3
	Scope for breaks/holidays (decide when to have a break / holidays)	2
	Meaning of work (perceiving work as meaningful / important)	2
	Commitment to the workplace/organisation (being proud to belong to this organisation)	2
**Social relations & leadership**	Predictability (being informed in advance about decisions, changes)	2
	Rewards (work is recognised and appreciated by one’s superior)	1
	Role clarity (clear work tasks, objectives, area of responsibility)	3
	Role conflicts (contradicting role requirements)	3
	Quality of leadership (superior is good at work planning, solving conflicts)	4
	Social support at work (support received from colleagues/superior)	4
	Feedback (feedback received from superior)	2
	Social relations at work (possibility to talk to colleagues during work)	1
	Social community (atmosphere, co-operation)	2
	Unfair behaviour / mobbing (feeling unjustly criticized by colleagues/superior)	1
**Person-work interface**	Job insecurity (worry about becoming unemployed)	4
	Insecurity of the working environment (changes in shift schedules)	2
**Home-work interface**	Work-private life conflict (conflict between work and private life)	5
	Demarcation (being available in leisure time for work issues)	2
**Stress symptoms & long-term consequences**	Behavioural stress symptoms (not having time to relax or enjoy life)	4
	Cognitive stress symptoms (problems concentrating, taking decisions)	4
	Job satisfaction (being pleased with work prospects, conditions)	6
	Intention to leave the organisation (thoughts on job changes)	1
	Intention to leave the profession (thoughts on career change)	1
	Burnout-symptoms (emotionally, physically exhausted)	3

The item responses are scored on a five-point Likert scale (1 = always, 2 = often, 3 = sometimes, 4 = seldom, 5 = never/hardly ever or 1 = to a very large extent, 2 = to a large extent, 3 = somewhat, 4 = to a small extent, 5 = to a very small extent). The polarity on the Likert scales differ between the scales, e.g. for scales on demands at work high scores indicate higher risk for work-related stress, while for the scales on opportunities for development or influence at work low scores indicate a higher risk for work-related stress. The total scale scores are arrived at based on average item-responses and transformed to a value range from 0 (never/hardly ever or to a very small extent) to 100 (always or to a large extent), taking account of reversed scored items as well. This transformation of items from 1 to 5 to 0-100 is done in most publications using the COPSOQ to allow comparability of results when using different COPSOQ Versions [22]. According to the original author of the COPSOQ [22], scale scores can be calculated if at least half the items are not missing (e.g. for a scale with 5 items, the mean is calculated if at least 3 of the 5 items are completed). No imputation procedure for missing values was performed.

### Translation and cultural adaption

**Fig. 1:**
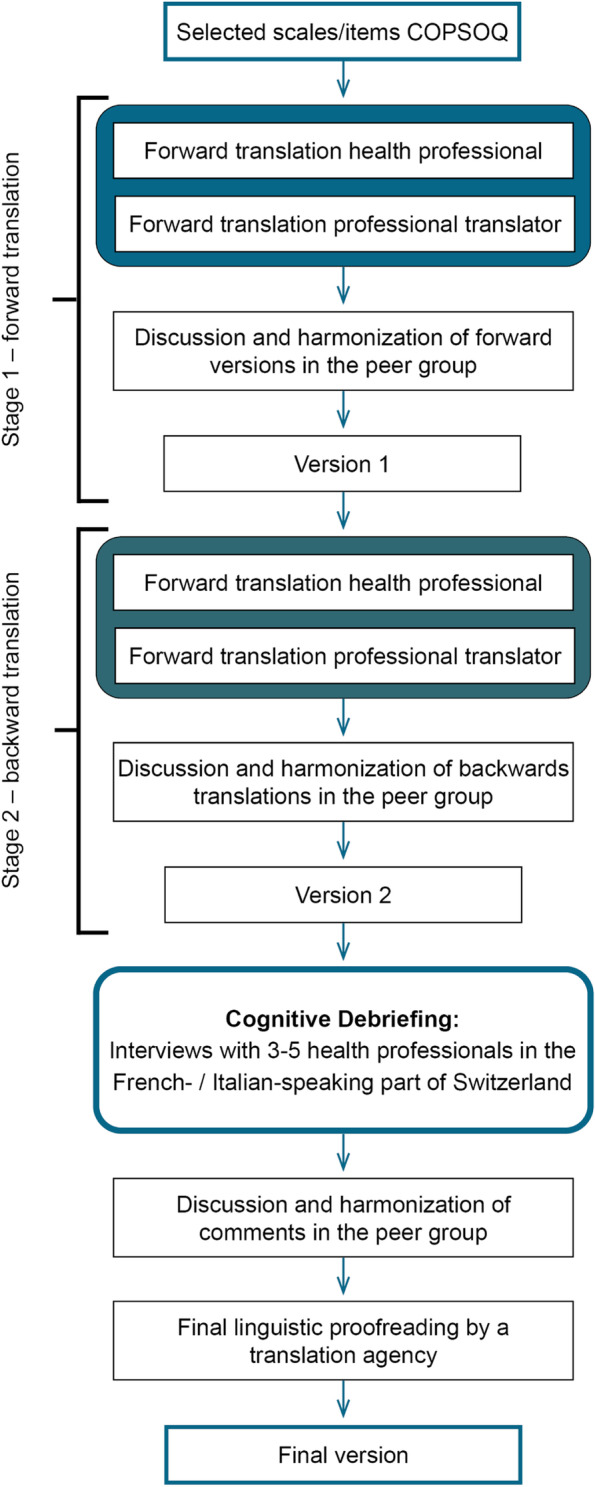
Methodological steps of translation and testing

Items from selected German-COPSOQ scales were translated and cross-culturally adapted to French and Italian in accordance with established guidelines for scientific translation processes “SPOR Principles of Good Practice” [35]. Figure 1 presents the stages of the translation process. In stage one, all items were independently forward translated by a native French/Italian-speaking health professional and a native French/Italian-speaking professional translator. After translation, the two versions were compared, discussed (peer group stage 1: two first authors and translators native French/Italian-speaking), and a common final version 1 was created. In stage two, the translated items were independently back translated into German by a French/Italian-speaking health professional and a translator, who were native German-speakers. Afterwards, language discrepancies were resolved by discussion (peer group stage 2: two first authors and translators native German-speaking), and a final version 2 was created. If questions arose regarding the comprehensibility of individual items, the original author of the German COPSOQ scale was involved. In a last step, the translated items were tested using ‘cognitive debriefing’ [35], to determine acceptability, understandability and clarity of translation. For this purpose, interviews with 5 native French-speaking and 5 native Italian-speaking health professionals were conducted and all items tested. After those interviews, a few adjustments were made in the translation-team (two first authors, native French/Italian-speaking, and German-speaking translators). Afterwards a final version was created and proofread by a translation agency (Final Version).

### Data collection

For data collection, all health professionals (nurses, midwives, medical-technical, medical-therapeutic professionals, physicians) in the participating organisations were invited to participate. The questionnaire was available in an online and paper version (including a direct reply envelope) in a German, French and Italian Version. The participation was on a voluntary basis for organisations as well as for health professionals and they had the option to choose the version of the questionnaire they preferred (online or paper version).

### Psychometric and statistical analysis

Participants’ characteristics and validation statistics for all scales were stratified by language groups. Since not all scales contain a sufficient number of items to calculate all psychometric coefficients (e.g. single-item scales), reliability was calculated only for scales with at least two items [36] and construct validity for scales with at least three items [37]. Reliability was investigated using Cronbach alpha and intraclass correlation coefficients. Although Cronbach alpha is an accurate estimate for two items, it may underestimate true reliability [36]. Floor and ceiling effects were calculated as the proportion of respondents choosing the lowest and highest response options for all items within a scale, adhering to the procedure from comparable studies [23, 38].

Furthermore, we calculated Intra Class Correlations (ICC) (3,1) in accordance with the recommendation by Shrout and Fleiss [39] that ICCs (3,1) be used to measure the consistency of multiple ratings (two-way mixed effects analysis of variance (ANOVA); each subject is measured by a fixed set of items), using the psych package in R [40]. For Cronbach Alpha, values > 0.7 indicate scale suitability, whereby a higher number of items normally results in a higher coefficient [41]. For ICC values, less than 0.4, between 0.4 and 0.59, between 0.60 and 0.74, and greater than 0.75 are indicative of poor, fair, good, and excellent reliability, respectively [42].

Construct validity and associations between latent constructs were estimated using confirmatory factor analysis (CFA) and structural equation modelling using latent variable analysis in R [43, 44]. CFA tests the given theoretical model and defines its measure of quality [45]. Construct validity was estimated a) on scale levels by using single items as indicators, and b) on domain levels by using the mean values of scales as indicators. For the latter we used structural equation modelling to assess the strength of association between the different psychological domains. Standardized loadings/coefficients (β), corresponding standard errors (S.E), and R-squared (amount of scale variance explained by latent variable) are shown. The values for factor loadings were seen as satisfactory above 0.4 [46]. Various measures were used to estimate model fit. A root mean-square error of approximation (RMSEA) below 0.05 was considered good (below 0.08 as acceptable); a Standardized Root Mean Square Residual (SRMR) below 0.08, and comparative fit index (CFI) above 0.95 were considered satisfactory fit [43, 47, 48]. In multilingual studies, comparability of the data from different language versions is crucial. Hence, the assumption that the instrument measures the same psychological construct across language groups was tested. To compare CFA models (on scale levels) across language groups, likelihood ratio tests were conducted [49]. Analyses were performed using R (version 3.5.1) [50].

## Results

### Study sample description

A total of 12,754 health professionals completed the questionnaire with a mean age of 41.48 years (SD 12.47). A total of 10,738 (84.2%) were German-, 1788 (14.0%) French-, and 228 (1.8%) Italian-speaking. Most of the respondents were female (81%), nurses (58%), and worked in the acute care setting (42.8%). Participants’ characteristics are shown in Supplement B. The percentage of missing values on scale level was between 7 and 13%. Most of the scales had low floor and ceiling effects, except for the scales “unfair behaviour”, “intention to leave the profession” and “intention to leave the organisation”.

### Reliability

Table 2 shows the results for reliability of the scales stratified by language group. Scales that include at least two items were considered for calculation. In the German version 20 of the 24 scales with at least two items exceeded the conventional threshold of 0.7 for Cronbach’s alpha, indicating sufficient internal consistency, whereas in the French version 19 and in the Italian version 17 reached the threshold of 0.7 for Cronbach’s alpha. The scales “Quantitative demands”, “Opportunities for development”, “Scope for breaks and holidays”, “Feedback”, and “Demarcation”, failed to show desirable levels for Cronbach’s alpha in some or in all language groups, ranging from 0.39 – 0.68. The vast majority of scales showed fair (0.40 - 0.59) or good (0.60 – 0.74) scale consistency as measured by ICC.

**Table 2:** Reliability of the German, French, and Italian of the modified COPSOQ scale

Scale Name (Number of items)	German Version						French Version						Italian Version					
	N	Mean (SD)	Floor %	Ceiling %	Alpha	ICC3	N	Mean (SD)	Floor %	Ceiling %	Alpha	ICC3	N	Mean (SD)	Floor %	Ceiling %	Alpha	ICC3
**Domain: Demands at work**																		
Quantitative demands (3)	9988	55.11 (17.36)	0.23	0.44	0.70	0.44	1628	56.53 (16.44)	0.18	0.43	0.62	0.34	221	48.59 (16.36)	0.90	0.00	0.56	0.29
Demands for hiding emotions (2)	9536	40.05 (22.27)	9.98	1.64	0.81	0.68	1507	41.74 (24.67)	8.16	3.45	0.76	0.61	224	35.66 (24.89)	12.95	3.13	0.71	0.57
Sensorial demands (5)	9978	83.75 (14.16)	0.01	19.08	0.78	0.40	1627	78.7 (17.17)	0.06	14.38	0.81	0.44	222	80.46 (16.45)	0.00	13.06	0.77	0.37
Work environment (5)	9899	32.95 (19.51)	3.94	0.15	0.75	0.38	1596	36.18 (20.07)	3.51	0.38	0.71	0.33	222	32.52 (20.57)	4.95	0.00	0.73	0.35
**Domain: Work organisation & content**																		
Opportunities for development (3)	10,034	71.15 (15.42)	0.07	5.10	0.68	0.41	1639	71.65 (17.23)	0.00	7.32	0.68	0.42	222	69.69 (17.47)	0.00	7.21	0.65	0.39
Influence at work (3)	9856	51.41 (20.07)	1.25	1.13	0.71	0.45	1596	47.72 (21.3)	2.38	1.38	0.74	0.48	222	53.83 (19.13)	3.60	6.31	0.75	0.49
Scope for breaks and holidays (2)	9871	60.75 (20.81)	1.01	4.86	0.39	0.24	1599	60.65 (21.91)	1.44	4.82	0.43	0.26	224	55.86 (25.11)	3.57	6.25	0.48	0.32
Meaning of work (2)	10,031	83.39 (15.95)	0.11	37.15	0.83	0.70	1641	82.63 (17.06)	0.12	35.71	0.79	0.66	221	82.07 (18.62)	1.36	22.17	0.81	0.69
Bond with the organisation (2)	9543	62.15 (19.99)	0.71	7.28	0.76	0.62	1512	61.11 (24.24)	1.46	12.70	0.77	0.63	224	68.25 (24.46)	1.34	21.88	0.87	0.77
**Social relations & leadership**																		
Predictability (2)	9811	64.46 (19.1)	0.41	6.49	0.71	0.54	1575	57.41 (22.58)	1.40	6.29	0.75	0.59	226	57.91 (23.34)	2.21	7.96	0.81	0.67
Appreciation (1)	9673	59.21 (25.52)	5.44	11.57	*n.a.*	*n.a.*	1501	48.72 (29.12)	12.72	9.19	*n.a.*	*n.a.*	223	65.02 (25.08)	2.69	20.18	*n.a.*	*n.a.*
Role clarity (3)	9815	77.82 (14.51)	0.05	14.37	0.79	0.55	1575	79.69 (17.18)	0.06	23.56	0.84	0.63	226	77.71 (17.7)	0.88	21.24	0.84	0.63
Role conflicts (3)	9798	36.74 (20.23)	6.40	0.66	0.79	0.55	1568	37.86 (22.78)	4.27	1.91	0.81	0.58	226	30.9 (24.57)	12.83	1.77	0.86	0.67
Quality of leadership (4)	9700	64.8 (22.07)	1.30	7.82	0.90	0.69	1555	59.51 (24.27)	2.32	6.70	0.90	0.68	222	58.19 (24.11)	1.80	8.56	0.91	0.71
Social support at work (4)	9678	77.14 (17.06)	0.07	15.69	0.81	0.52	1549	72.28 (18.25)	0.06	9.94	0.80	0.51	224	74.56 (18.66)	0.00	13.84	0.85	0.58
Feedback (2)	9668	49.93 (20.56)	2.29	1.72	0.62	0.45	1544	50.99 (22.86)	2.53	4.02	0.65	0.48	224	64.68 (20.71)	1.79	8.04	0.65	0.47
Social relations at work (1)	9560	56.16 (26.79)	8.45	10.37	*n.a.*	*n.a.*	1536	68.12 (25.62)	4.23	23.18	*n.a.*	*n.a.*	222	73.42 (22.51)	1.80	8.11	*n.a.*	*n.a.*
Social community at work (2)	9646	79.59 (14.62)	0.16	22.49	0.83	0.71	1545	79.64 (15.75)	0.13	25.95	0.85	0.74	224	79.07 (18.25)	0.00	29.02	0.87	0.76
Unfair behaviour (1)	9437	14.08 (21.52)	63.37	0.58	*n.a.*	*n.a.*	1497	14.43 (21.82)	62.86	0.73	*n.a.*	*n.a.*	220	15.11 (23.2)	0.00	29.55	*n.a.*	*n.a.*
**Person-work interface**																		
Job insecurity (4)	6245	16.9 (17.32)	28.52	0.13	0.76	0.43	1018	30.63 (23.78)	13.56	0.79	0.79	0.49	185	26.96 (16.31)	6.49	0.00	0.56	0.23
Insecurity of working environment (2)	9896	27.1 (23.54)	24.90	1.50	0.71	0.55	1602	44.46 (30.29)	13.80	7.18	0.69	0.53	224	30.13 (24.61)	20.98	2.23	0.59	0.42
**Home-work interface**																		
Work-private life conflict (5)	9619	28.16 (20.81)	11.72	0.32	0.89	0.61	1519	32.62 (22.76)	9.28	0.53	0.88	0.60	224	34.67 (21.52)	3.13	0.45	0.88	0.59
Demarcation (2)	9618	32.25 (21.7)	13.84	0.75	0.39	0.24	1517	30.96 (24.1)	20.44	1.32	0.40	0.24	224	30.41 (22.51)	17.86	1.79	0.41	0.25
**Stress symptoms & long-term consequences**																		
Behavioural stress symptoms (4)	9468	29.29 (21.68)	7.46	0.07	0.86	0.61	1483	31.27 (23.17)	5.60	0.27	0.86	0.61	225	34.24 (21.28)	6.67	0.00	0.83	0.55
Cognitive stress symptoms (4)	9486	25.75 (19.04)	15.75	0.14	0.89	0.66	1490	32.86 (21.74)	11.68	0.34	0.91	0.72	225	29.57 (20.29)	15.56	0.00	0.90	0.69
Job satisfaction (6)	9545	70.61 (14.41)	0.05	2.76	0.81	0.42	1504	66.82 (15.81)	0.00	2.53	0.84	0.45	225	67.4 (17.25)	1.33	4.44	0.89	0.58
Intention to leave organisation (1)	9501	20.6 (22.82)	53.65	1.00	*n.a.*	*n.a.*	1501	19.6 (23.2)	56.90	1.13	*n.a.*	*n.a.*	225	14.11 (20.68)	64.44	0.89	*n.a.*	*n.a.*
Intention to leave profession (1)	9501	16.26 (21.33)	42.95	1.43	*n.a.*	*n.a.*	1501	15.39 (21.42)	46.64	1.53	*n.a.*	*n.a.*	225	11.89 (18.91)	59.56	1.33	*n.a.*	*n.a.*
Burnout-symptoms (3)	9407	41.53 (20.81)	4.23	0.82	0.84	0.63	1463	49.69 (20.41)	1.71	1.37	0.83	0.62	225	36.56 (21.56)	8.44	0.44	0.82	0.60

*Alpha* Cronbach’s coefficient α, *ICC3* Consistency estimate by two-way mixed effects ANOVA

### Validity

**Fig. 2:**
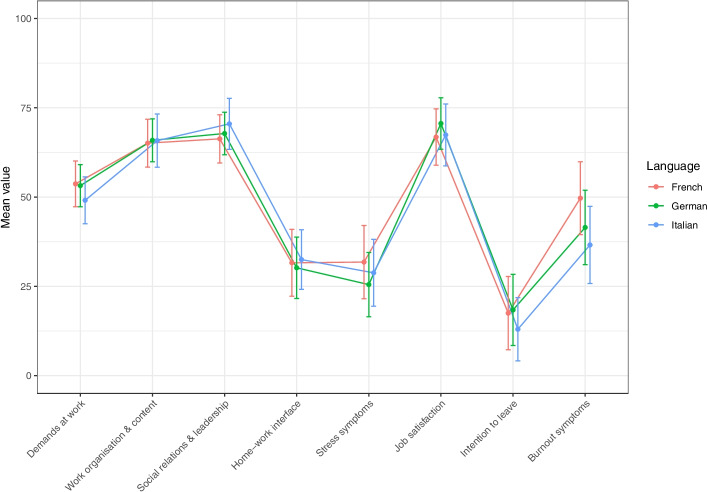
Graphic comparison of mean values and standard deviation (SD) from the German, French and Italian version. Mean values and SD for demands at work, work organisation & content, social relations & leadership, home-work interface, stress symptoms, job satisfaction, intention to leave, burnout symptoms (all standard deviations are overlapping)

Figure 2 illustrates the mean values (between 0 and 100) on the domain level (demands at work, work organisation & content, social relations & leadership, home-work interface and stress symptoms) as well as scales on job satisfaction, intention to leave (the organisation / the profession) and burnout symptoms. The figure demonstrates that the mean values for the German, French and Italian versions show similar low or high relative tendencies for each dimension/scale.

### Construct validity on scale level

In Table 3 the results of the CFA for each scale by language using single items as indicators are presented. R-squared showed predominantly satisfactory factor loadings with values higher than 0.40 in all language groups. In Table 4 the corresponding results from the estimate model fit for each scale and language version are presented. The majority of the scales indicated a good to satisfactory fit with an RMSEA below 0.1, SRMR below 0.08 and CFI above 0.95. The scale Social Support at work could not meet any of the criteria in any language versions.

**Table 3:** Results for the confirmatory factor analysis by scale including loadings, standard errors and variance explained, stratified by language

Scales (> 2 items)	German			French			Italian		
	Estimate (stand. β)	SE	R2	Estimate (stand. β)	SE	R2	Estimate (stand. β)	SE	R2
**Demands at work**									
Quantitative demands (QD)									
QD1	0.65	0.01	0.42	0.60	0.03	0.36	0.46	0.09	0.21
QD2	0.77	0.01	0.59	0.73	0.03	0.54	0.84	0.14	0.70
QD3	0.58	0.01	0.33	0.46	0.03	0.21	0.39	0.09	0.15
Sensorial demands (SD)									
SD1	0.57	0.01	0.32	0.64	0.02	0.41	0.46	0.06	0.21
SD2	0.70	0.01	0.49	0.63	0.02	0.40	0.61	0.05	0.37
SD3	0.61	0.01	0.38	0.61	0.02	0.37	0.61	0.05	0.37
SD4	0.67	0.01	0.45	0.71	0.02	0.50	0.76	0.04	0.58
SD5	0.68	0.01	0.46	0.80	0.01	0.63	0.74	0.04	0.55
Work environment (WE)									
WE1	0.50	0.01	0.25	0.53	0.02	0.28	0.55	0.06	0.30
WE2	0.52	0.01	0.27	0.55	0.02	0.30	0.44	0.07	0.19
WE3	0.71	0.01	0.51	0.69	0.02	0.47	0.69	0.05	0.47
WE4	0.69	0.01	0.47	0.56	0.02	0.32	0.59	0.06	0.34
WE5	0.66	0.01	0.44	0.61	0.02	0.37	0.71	0.05	0.51
**Work organisation & content**									
Opportunities for development (OD)									
OD1	0.55	0.01	0.30	0.49	0.02	0.24	0.31	0.07	0.10
OD2	0.69	0.01	0.48	0.79	0.03	0.63	0.94	0.11	0.89
OD3	0.69	0.01	0.48	0.67	0.03	0.45	0.69	0.09	0.48
Influence at work (INF)									
INF1	0.62	0.01	0.38	0.70	0.02	0.49	0.85	0.05	0.73
INF2	0.70	0.01	0.49	0.66	0.02	0.44	0.55	0.06	0.30
INF3	0.70	0.01	0.49	0.74	0.02	0.54	0.74	0.05	0.55
**Social relations & leadership**									
Role clarity (RCL)									
RCL1	0.60	0.01	0.36	0.67	0.02	0.45	0.69	0.04	0.47
RCL2	0.82	0.01	0.67	0.90	0.01	0.81	0.87	0.03	0.75
RCL3	0.82	0.01	0.66	0.83	0.01	0.69	0.86	0.03	0.73
Role conflicts (RCF)									
RCF1	0.66	0.01	0.43	0.70	0.02	0.49	0.77	0.03	0.59
RCF2	0.88	0.01	0.77	0.84	0.02	0.71	0.92	0.03	0.84
RCF3	0.71	0.01	0.50	0.74	0.02	0.55	0.79	0.03	0.62
Quality of leadership (QOL)									
QOL1	0.82	0.00	0.67	0.81	0.01	0.66	0.85	0.02	0.72
QOL2	0.88	0.00	0.77	0.89	0.01	0.79	0.90	0.02	0.82
QOL3	0.80	0.00	0.65	0.80	0.01	0.64	0.79	0.03	0.62
QOL4	0.84	0.00	0.70	0.81	0.01	0.66	0.83	0.03	0.68
Social support at work (SOS)									
SOS1	0.54	0.01	0.29	0.47	0.02	0.22	0.77	0.04	0.59
SOS2	0.56	0.01	0.31	0.52	0.02	0.27	0.86	0.03	0.74
SOS3	0.88	0.00	0.78	0.91	0.01	0.84	0.69	0.04	0.48
SOS4	0.84	0.01	0.70	0.85	0.01	0.72	0.74	0.04	0.54
**Person-work interface**									
Job insecurity (JIS)									
JIS1	0.84	0.01	0.71	0.83	0.02	0.69	0.57	0.09	0.33
JIS2	0.57	0.24	0.32	0.61	0.02	0.37	0.55	0.09	0.30
JIS3	0.69	0.35	0.48	0.77	0.02	0.59	0.51	0.09	0.26
JIS4	0.56	0.31	0.32	0.61	0.02	0.37	0.35	0.09	0.12
**Home-work interface**									
Work-private life conflict (WPC)									
WPC1	0.79	0.00	0.63	0.75	0.01	0.56	0.71	0.04	0.50
WPC2	0.85	0.00	0.72	0.81	0.01	0.65	0.79	0.03	0.63
WPC3	0.80	0.00	0.64	0.86	0.01	0.73	0.81	0.03	0.66
WPC4	0.89	0.00	0.79	0.88	0.01	0.78	0.88	0.02	0.77
WPC5	0.59	0.01	0.35	0.56	0.02	0.32	0.63	0.04	0.40
**Stress symptoms & long-term consequences**									
Behavioural stress symptoms (BSS)									
BSS1	0.78	0.01	0.61	0.71	0.01	0.59	0.73	0.04	0.54
BSS2	0.74	0.01	0.55	0.71	0.02	0.37	0.67	0.04	0.45
BSS3	0.80	0.01	0.64	0.82	0.01	0.36	0.73	0.04	0.53
BSS4	0.80	0.00	0.64	0.89	0.01	0.54	0.83	0.03	0.69
Cognitive stress symptoms (CSS)									
CSS1	0.80	0.00	0.65	0.86	0.01	0.74	0.84	0.02	0.71
CSS2	0.75	0.01	0.56	0.79	0.01	0.63	0.79	0.03	0.62
CSS3	0.82	0.00	0.67	0.86	0.01	0.73	0.82	0.03	0.67
CSS4	0.88	0.00	0.78	0.89	0.01	0.80	0.87	0.02	0.76
Job satisfaction (JSA)									
JSA1	0.57	0.01	0.33	0.60	0.02	0.37	0.80	0.03	0.64
JSA2	0.58	0.01	0.33	0.56	0.02	0.32	0.61	0.05	0.37
JSA3	0.46	0.01	0.21	0.59	0.02	0.35	0.69	0.04	0.47
JSA4	0.74	0.01	0.54	0.76	0.01	0.57	0.79	0.03	0.62
JSA5	0.78	0.01	0.61	0.76	0.01	0.58	0.84	0.02	0.71
JSA6	0.78	0.01	0.61	0.80	0.01	0.65	0.84	0.02	0.70
Burnout-symptoms (BUS)									
BUS1	0.88	0.01	0.77	0.85	0.01	0.71	0.76	0.04	0.57
BUS2	0.83	0.01	0.68	0.80	0.01	0.64	0.87	0.04	0.76
BUS3	0.69	0.01	0.47	0.72	0.02	0.52	0.70	0.04	0.49

Included are scales > 2 items, *n.a.* CFA not applicable (too few indicators), *Estimate (stand. β)* Standardized loadings/coefficients, *SE* Standard errors, *R2* R-squared

**Table 4:** FIT measures of scales by language

Scales	Language	Fit measures		
		RMSEA	SRMR	CFI
**Demands at work**				
Quantitative demands	German	0.00	0.00	1.00
	French	0.00	0.00	1.00
	Italian	0.00	0.00	1.00
Sensorial demands	German	0.14	0.05	0.92
	French	0.12	0.04	0.96
	Italian	0.13	0.05	0.93
Work environment	German	0.09	0.03	0.97
	French	0.09	0.04	0.95
	Italian	0.10	0.05	0.94
**Work organisation & content**				
Opportunities for development	German	0.00	0.00	1.00
	French	0.00	0.00	1.00
	Italian	0.00	0.00	1.00
Influence at work	German	0.00	0.00	1.00
	French	0.00	0.00	1.00
	Italian	0.00	0.00	1.00
**Social relations & leadership**				
Role clarity	German	0.00	0.00	1.00
	French	0.00	0.00	1.00
	Italian	0.00	0.00	1.00
Role conflicts	German	0.00	0.00	1.00
	French	0.00	0.00	1.00
	Italian	0.00	0.00	1.00
Quality of leadership	German	0.10	0.01	0.99
	French	0.15	0.02	0.98
	Italian	0.21	0.03	0.97
Social support at work	German	0.36	0.10	0.84
	French	0.38	0.12	0.83
	Italian	0.45	0.09	0.80
**Person-work interface**				
Job insecurity	German	0.07	0.02	0.99
	French	0.07	0.02	0.99
	Italian	0.10	0.04	0.94
**Home-work interface**				
Work-private life conflict	German	0.15	0.03	0.96
	French	0.19	0.04	0.94
	Italian	0.15	0.04	0.95
**Stress symptoms & long-term consequences**				
Behavioural stress symptoms	German	0.18	0.03	0.97
	French	0.14	0.03	0.98
	Italian	0.17	0.04	0.96
Cognitive stress symptoms	German	0.11	0.02	0.99
	French	0.10	0.01	0.99
	Italian	0.19	0.03	0.97
Job satisfaction	German	0.07	0.03	0.97
	French	0.08	0.03	0.97
	Italian	0.11	0.04	0.96
Burnout-symptoms	German	0.00	0.00	1.00
	French	0.00	0.00	1.00
	Italian	0.00	0.00	1.00

*n.a.* CFA not applicable (too few indicators), *CFI* Comparative Fit Index, *RMSEA* Root Mean Square Error of Approximation, *SRMR* Standardized Root Mean Square Residual

### Factor invariance

The measurement of invariance tests the psychometric equivalence of the construct across groups. Table 5 presents the findings of the invariance test. The test for factor invariance indicates a variance across the language versions with *p*-values of < 0.05. For 10 out of 15 scales a significant difference regarding the psychological construct across the language versions is expected. All dimensions included scales, which showed variance across language versions. In particular, the dimensions Work organisation & content as well as Home-work interface comprised solely of scales with variance across the languages.

**Table 5:** Test of factor invariance (loadings confirmatory factor analysis) across multiple across language groups

Scales	X2 difference in loadings	Difference in loadings	***P***-value
**Demands at work**			
Quantitative demands	8.89	4	0.0638
Sensorial demands	100.66	8	*p* < 0.001
Work environment	63.73	8	*p* < 0.001
**Work organisation & content**			
Opportunities for development	21.25	4	0.0003
Influence at work	23.10	4	0.0001
**Social relations & leadership**			
Role clarity	10.45	4	0.0334
Role conflicts	11.21	4	0.0243
Quality of leadership	12.45	6	0.0526
Social support at work	43.47	6	*p* < 0.001
**Person-work interface**			
Job insecurity	3.23	6	0.7801
**Home-work interface**			
Work-private life conflict	37.68	8	*p* < 0.001
**Stress symptoms & long-term consequences**			
Behavioural stress symptoms	99.22	6	*p* < 0.001
Cognitive stress symptoms	5.26	6	0.5108
Job satisfaction	65.01	10	*p* < 0.001
Burnout-symptoms	6.19	4	0.1857

*P*-values of < 0.05 indicate evidence of loading variance across the German, French, and Italian versions of the scale

### Construct validity on dimension level

**Fig. 3:**
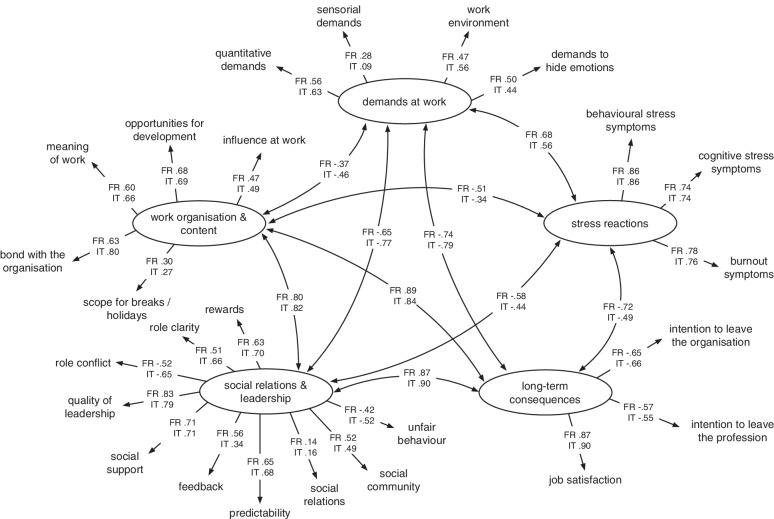
Structural equation models on dimension and scale level. Structural equation models using dimensions as latent constructs and scales as indicators in the French (FR, *n* = 1788) and Italian group (IT, *n* = 228), respectively

Figure 3 summarizes the relationships between the dimensions and the assigned scales for the French and Italian versions. Models show that the majority of indicators show strong relationships with its dimensions except for social relations (both languages) and sensorial demands (Italian group). The majority of the latent dimensions for the French version are strongly interrelated ranging from − 0.65 - -0.72 as well as positive relations ranging from 0.68 – 0.89. In the Italian version, half of the latent dimensions show medium interrelations with − 0.34 - -0.49, respectively 0.56, and the other half of the latent dimensions show strong interrelations with − 0.77, respectively 0.79 – 0.9.

Model fit was acceptable for RMSEA (FR 0.08, IT 0.08), and SRMR (FR 0.07, IT 0.07), respectively. Models did not show a satisfactory fit with regards to CFI (FR 0.82, IT 0.82) in either language.

## Discussion

Valid versions of the COPSOQ are already available in the languages German [25, 26], French [27] and Italian [28]. However, for the first time, a questionnaire for measuring stressors and consequences of work-related stress among health professionals is available for multilingual studies in the three languages German, French and Italian which is, to some extent, comparable across those languages. Most of the translated and tested scales showed acceptable to good internal consistency. The CFA tends to verify the underlying theoretical model of Nübling, Stößel [25], which has been already tested for concurrent validity [51]. It also confirms the strong relationships between the dimensions, as well as the low values for the scales social relations and sensorial demands; we therefore underline the proposition to remove or revise those scales [21].

Moreover, the results are comparable to a recently published study in which the latest version of the underlying questionnaire (COPSOQ III) was validated without an Italian version for international comparability [29]. However, there are differences regarding the reliability of some scales. In Burr, Berthelsen [29], the scales Predictability (0.62), Meaning of Work (0.62) and Job Insecurity (0.66) are given a below-threshold value of 0.7, whereas in this study the scales Quantitative Demands (0.56 - 0.62), Opportunities for Development (0.65 - 0.68), Scope for breaks and holidays (0.39 - 0.43), Feedback (0.62 - 0.65) and Demarcation (0.39 - 0.40) were revealed to be unsatisfactory in terms of achieving the threshold. However, the scales for Feedback and Demarcation are no longer included in the COPSOQ III, which makes comparison of those two scales with the study of Burr, Berthelsen [29] impossible and highlights the diversity of the included scales within the national versions. Hence, the scales Feedback and Demarcation can be excluded in accordance with the latest COPSOQ III version. Furthermore, the COPSOQ III has the dimension Control over Working Time included, which consists of 4 items with a Cronbach’s alpha of 0.69 [28]. Two items match with the items of the Scale Scope for breaks and holidays, which was found to have a low reliability in this study as well as the study evaluating the German COPSOQ version [52]. The authors of the COPSOQ German version have acknowledged this issue and stated to observe it in further studies [52]. In the meantime, pending further development of the COPSOQ by the responsible COPSOQ network, researchers must decide in each case when using the current version as to whether international comparability or reliability is prioritised. When deciding for international comparability, it should be noted that the reliability of comparability would be limited.

Furthermore, the data used in the study of Burr, Berthelsen [29] are company-specific and collected across a multitude of branches, whereas in this study the data comes from health professionals working in the healthcare system, and are thus expected to differ to a large extent with regard to the working conditions and occupational culture.

Independently of the language version, short scales were affected by lower reliabilities. This finding might contribute to the discussed dependency of Cronbach’s alpha on the number of items [53]. In addition, some findings imply the evaluation of the scales, whether they should be enriched with additional items or excluded from the questionnaire.

Cultural and regional differences may have led to the different reliability per scale across language versions and therefore to a significant factor variance in 10 out of 15 scales. Although the variances have been demonstrated statistically, the question arises as to their clinical relevance. The differences in the estimates from Table 3 across the language versions aggregated on the scale level could indicate what statistically significant variance can nonetheless be tolerated for comparability across languages. Of the 10 scales with significant factor variance, four showed a difference > 0.1 in the estimates (opportunities for development, influence at work, social support at work, job satisfaction), implying a revision of those scales to enhance comparability across language versions. In particular, the scale social support at work showed unsatisfactory FIT measures with RMSEA > 0.05, SRMR > 0.08 and CFI < 0.95. Unfortunately, FIT measures on scale level of the COPSOQ from other studies are not available for comparison [28]. In this respect, there is a particular need for a revision of this scale in terms of correct translation and fit. In addition, future studies should include FIT measures in the psychometric testing of the COPSOQ. When using the current version, one should not assign too much significance to the results of the scale social support at work. In Switzerland researchers have to deal with a heterogenous population when surveying nationally, due to the different language regions, despite the country’s small size in relation to other countries. It is known that linguistic differences often go hand in hand with cultural differences and therefore should be considered when developing a measurement across languages and/or cultures [54]. Several questionnaires appeared to struggle with invariance across language versions [30]. One reason for the statistical differences across the language versions could be that the French and Italian language regions in Switzerland have higher numbers of foreign health professionals, such as cross-border workers [55], whose evaluation criteria might differ from those of domestic personnel, for example in terms of job insecurity (e.g. migration policy). An analysis of the missings at the item level could indicate cultural issues, which should be addressed in order to enhance comparability.

Moreover, the enormous change in healthcare systems brought about by digitization [56] implies the emergence of new influencing factors from the interaction of health professionals with technology. However, new trends are continuously being monitored by the COPSOQ international network and, are thus being incorporated into the further development of the COPSOQ [29].

## Strengths & limitations

Besides a structured and carefully implemented translation process, one strength of the study is the large sample size across all health professions, settings and language regions, which allows a generalization of the findings. This study delivers important information for further research enabling multilingual research in measuring stressors and consequences of stress at work among health professionals in Switzerland. It provides an extensive amount of information on scales, which is expected to be helpful in future research aimed at advancing scale development and choosing appropriate scales. For the first time, language versions of the COPSOQ were comprehensively statistically analysed for their consistent measurement of the underlying construct.

Although the strengths are promising, they must be considered in the context of the limitations, since two-thirds of the scales differ significantly regarding the measured psychological construct in the language versions. In addition, the results presented in this study are limited to the healthcare sector. Therefore, further psychometric testing of the new multilingual COPSOQ Versions in Italian and French should be carried out in other work sectors to further confirm our results. Hence, interpretation of the results across language regions must be made in the context of these differences. The findings could have originated in the bottom or ceiling effects that were identified, which indicate limited discrimination properties of some scales. Moreover, the study included data sets from two measurement periods, which may have led to duplicates, and, in turn, to cases of duplicates remaining undetected due to possible misstatements. Future research should allow to assign two measurement points to one individual, which would enable to conduct an analysis of test-retest reliability. This analysis has been found to be more appropriate for the analysis of the reliability of psychosocial work environment scales [57]. Finally, several scales were measured with single-items or two items; it is thus possible that the construct to be measured was not sufficiently covered by these items.

## Conclusions

This article presents the psychometric properties of a trilingual questionnaire that measures stressors and consequences of stress at work among health professionals. The COPSOQ is known as a generic instrument across branches. An adaptation to working conditions in the healthcare sector could optimize the psychometric properties of the instrument. Hence, future investigation to optimize internal and construct validity of some scales and dimensions is needed to improve the questionnaire. The identified variances across language versions imply re-evaluating the questionnaire to determine whether it is biased by cultural factors, which should be identified in advance.

## Supplementary Information

**Additional file 1: Supplement A.** Original COPSOQ-items (English and German) and translated COPSOQ-items in this study (French and Italian).

**Additional file 2: Supplement B.** Participants and setting characteristics stratified by language (German, French, Italian).

## Data Availability

The raw data was generated at the Bern University of Applied Sciences, Department of Nursing Research. Derived data supporting the findings of this study is available from the corresponding author (karin.peter@bfh.ch) upon request.

## Abbreviations

ANOVA: Analysis of variance
CFA: Confirmatory factor analysis
COPSOQ: Copenhagen Psychosocial Questionnaire
ICC: Intra Class Correlations
RMSEA: Root mean-square error of approximation
SRMR: Standardized Root Mean Square Residual
STRAIN: Work-related stress among health professionals in Switzerland
