# Supplementary material for: Assessing the psychosocial work environment in the health care setting: translation and psychometric testing of the French and Italian Copenhagen Psychosocial Questionnaires (COPSOQ) in a large sample of health professionals in Switzerland
Source: BMC Health Serv Res. 2022 May 6;22:608. doi: 10.1186/s12913-022-07924-4 (PMC9074249; doi:10.1186/s12913-022-07924-4)
Supplement: Supplementary file 1 — Additional file 1: Supplement A. Original COPSOQ-items (English and German) and translated COPSOQ-items in this study (French and Italian). [file 12913_2022_7924_MOESM1_ESM.docx]

**Supplement A:** Original COPSOQ-items (English and German) and translated COPSOQ-items in this study (French and Italian**)**

|  | **Original Items in English** |  | **Original Items in German** |  | **Translated Items - French** |  | **Translated Items - Italian** |
| --- | --- | --- | --- | --- | --- | --- | --- |
|  |  | | | | |  |  |
| **Demands at work** | | | | | | | |
| Quantitative demands (QD) | | | | | | | |
| QD1 | Do you have to work very fast? |  | Müssen Sie sehr schnell arbeiten? |  | Devez-vous travailler très vite? |  | Deve lavorare molto velocemente? |
| QD2 | How often do you not have time to complete all your work tasks? |  | Wie oft kommt es vor, dass Sie nicht genügend Zeit haben, alle Ihre Aufgaben zu erledigen? |  | À quelle fréquence vous arrive t-il de ne pas avoir le temps d'effectuer toutes vos tâches? |  | Quanto spesso non ha tempo di portare a termine tutti i compiti connessi col Suo lavoro? |
| QD3 | Do you have to do overtime? |  | Müssen Sie Überstunden machen? |  | Etes-vous amené-e à faire des heures supplémentaires? |  | Deve fare straordinari/lavoro extra? |
|  | | | | | | | |
| Demands to hide emotions (DHE) | | | | | | | |
| DHE1 | Does your work require that you hide your feelings? |  | Verlangt Ihre Arbeit von Ihnen, dass Sie Ihre Gefühle verbergen? |  | Votre travail exige-t-il que vous dissimuliez vos sentiments? |  | Il Suo lavoro esige che Lei nasconda i Suoi emozioni? |
| DHE2 | Does your work require that you do not state your opinion? |  | Verlangt Ihre Arbeit von Ihnen, sich mit Ihrer Meinung zurück zu halten? |  | Votre travail exige-t-il que vous n'exprimiez pas votre opinion? |  | Il suo lavoro esige che Lei non espliciti le Sue opinioni? |
|  |  | | | | |  |  |
| Sensorial demands | | | | | | | |
| SD1 | Does your work demand a great deal of concentration? |  | Erfordert Ihre Arbeit ein hohes Mass an Konzentration? |  | Votre travail nécessite-t-il un grand degré de concentration? |  | Il Suo lavoro richiede intensi livelli di concentrazione? |
| SD2 | Does your work require that you have very clear and precise eyesight? |  | Erfordert Ihre Tätigkeit gutes und klares Sehvermögen? |  | Votre activité exige-t-elle une vue particulièrement bonne et nette? |  | Il Suo lavoro richiede una vista buona e chiara? |
| SD3 | Does your work require that you have to control your movements, e.g. your arms and hands consciously? |  | Erfordert Ihre Arbeit, Ihre Bewegungen, z.B. Arme und Hände, bewusst zu steuern? |  | Votre travail exige-t-il un contrôle particulier de vos mouvements, p.ex. des bras et des mains? |  | Il Suo lavoro richiede movimenti controllati degli arti? Per esempio delle braccia o delle mani? |
| SD4 | Does your work demand your constant attention? |  | Erfordert Ihre Arbeit ständige Aufmerksamkeit? |  | Votre travail nécessite-t-il une attention permanente? |  | Il Suo lavoro richiede continua attenzione? |
| SD5 | Does your work require a high level of precision? |  | Erfordert Ihre Arbeit einen hohen Grad an Genauigkeit? |  | Votre travail requiert-il un degré élevé de précision? |  | Il Suo lavoro richiede un alto livello di precisione? |
|  | | | | | | | |
| Work environment | | | | | | | |
| WE1 | not available in English |  | Wie häufig sind Sie an Ihrem Arbeitsplatz Lärm oder lauten Umgebungsgeräuschen ausgesetzt? |  | A quelle fréquence êtes-vous exposé-e au bruit ou à un bruit ambiant élevé à votre place de travail? |  | " Quanto spesso è esposto(a) al rumore o a un forte rumore ambientale sul Suo posto di lavoro?" |
| WE2 | not available in English |  | Wie häufig kommen Sie bei Ihrer Arbeit mit Chemikalien bzw. Gefahrstoffen in Berührung? |  | À quelle fréquence êtes-vous en contact avec des produits chimiques et/ou des substances dangereuses dans le cadre de votre travail? |  | Quanto spesso è in contatto con agenti chimici o pericolosi, sul Suo posto di lavoro? |
| WE3 | not available in English |  | Wie häufig sind Sie an Ihrem Arbeitsplatz Zugluft bzw. extremen Temperaturen ausgesetzt? |  | A quelle fréquence êtes-vous confronté-e à des courants d'air, ou des températures extrêmes à votre place de travail? |  | Quanto spesso viene esposto(a) a correnti d'aria o a temperature estreme, sul Suo posto di lavoro? |
| WE4 | not available in English |  | Wie häufig sind Sie an Ihrem Arbeitsplatz schlechter Luft ausgesetzt, z.B. Zigarettenrauch, Gasen oder Ähnlichem? |  | A quelle fréquence êtes-vous confronté-e à un air vicié, par ex. fumée de cigarette, gaz ou autres à votre place de travail? |  | Quanto spesso è esposto(a) ad aria nociva, per esempio fumo di sigarette, gas o sostanze simili, sul Suo posto di lavoro? |
| WE5 | not available in English |  | Wie häufig sind Sie bei der Arbeit schlechten Lichtverhältnissen ausgesetzt, z. B. grelles oder schwaches Licht? |  | A quelle fréquence êtes-vous confronté-e à une mauvaise lumière, par ex. lumière vive ou faible, à votre place de travail? |  | Quanto spesso è esposto(a) ad insufficiente illuminazione, per esempio a della luce fievole, sul Suo posto di lavoro ? |
|  | | | | | | | |
| **Work organisation and content** | | | | | | | |
| Opportunities for development (OD) | | | | | | | |
| OD1 | Is your work varied? |  | Ist Ihre Arbeit abwechslungsreich? |  | Votre travail est-il varié? |  | Il Suo lavoro è vario? |
| OD2 | Do you have the possibility of learning new things through your work? |  | Haben Sie die Möglichkeit, durch Ihre Arbeit neue Dinge zu erlernen? |  | Avez-vous la possibilité d’apprendre des choses nouvelles au travers de votre travail? |  | Ha la possibilità di apprendere cose nuove attraverso il Suo lavoro? |
| OD3 | Can you use your skills or expertise in your work? |  | Können Sie Ihre Fertigkeiten oder Ihr Fachwissen bei Ihrer Arbeit anwenden? |  | Pouvez-vous utiliser vos compétences ou votre expertise dans votre travail? |  | Può usare le Sue abilità o competenze nel Suo lavoro? |
|  |  | | | | |  |  |
| Influence at work (INF) | | | | | | | |
| INF1 | Do you have a large degree of influence concerning your work? |  | Haben Sie grossen Einfluss auf Entscheidungen, die Ihre Arbeit betreffen? |  | Avez-vous beaucoup d'influence sur les décisions qui concernent votre travail? |  | Quanta influenza ha sulle decisioni che riguardano il Suo lavoro? |
| INF2 | Can you influence the amount of work assigned to you? |  | Haben Sie Einfluss auf die Menge der Arbeit, die Ihnen übertragen wird? |  | Avez-vous une influence sur la quantité de travail qui vous est attribuée? |  | Può influenzare il quantitativo di lavoro assegnatole? |
| INF3 | Do you have any influence on what you do at work? |  | Haben Sie Einfluss darauf, was Sie bei Ihrer Arbeit tun? |  | Avez-vous une influence sur ce que vous faites dans votre travail? |  | Ha qualche influenza su quello che fa sul lavoro? |
|  | | | | | | | |
| Scope for breaks and holidays (SCO) | | | | | | | |
| SCO1 | Can you decide when to take a break? |  | Können Sie selbst bestimmen, wann Sie eine Pause machen? |  | Pouvez-vous déterminer vous-même quand vous effectuez une pause? |  | Può decidere da solo(a) quando fare pausa? |
| SCO2 | Can you take holidays more or less when you wish? |  | Können Sie mehr oder weniger frei entscheiden, wann Sie Urlaub machen? |  | Pouvez-vous plus ou moins librement décider quand vous prenez des vacances? |  | Può decidere più o meno in modo indipendente quando fare le vacanze? |
|  |  |  |  |  |  |  |  |
| Meaning of work (MOW) | | | | | | | |
| MOW1 | Is your work meaningful? |  | Ist Ihre Arbeit sinnvoll? |  | Votre travail a-t-il du sens pour vous? |  | Il Suo lavoro ha un senso? |
| MOW2 | Do you feel that the work you do is important? |  | Haben Sie das Gefühl, dass Ihre Arbeit wichtig ist? |  | Avez-vous le sentiment que votre travail est important? |  | Sente che il lavoro che fa è importante? |
|  |  |  |  |  |  |  |  |
| Commitment to the workplace/organisation (COM) | | | | | | | |
| COM1 | not available in English |  | Sind Sie stolz dieser Einrichtung anzugehören? |  | Êtes-vous fier(ère) de faire partie de votre établissement? |  | È orgoglioso(a) di far parte di questa organizzazione? |
| COM2 | Do you enjoy telling others about your place of work? |  | Erzählen Sie anderen gerne über Ihren Arbeitsplatz? |  | Aimez-vous parler aux autres de votre lieu de travail? |  | Le fa piacere parlare agli altri del Suo posto di lavoro? |
|  |  |  |  |  |  |  |  |
| **Social relations and leadership** | | | | | | | |
| Predictability (PRE) | | | | | | | |
|  |  |  |  |  |  |  |  |
| PRE1 | At your place of work, are you informed well in advance concerning for example important decisions, changes, or plans for the future? |  | Werden Sie rechtzeitig im Voraus über Veränderungen an Ihrem Arbeitsplatz informiert, z.B. über wichtige Entscheidungen oder Pläne für die Zukunft? |  | Au travail, êtes-vous informé(e) suffisamment à l’avance, p. ex. à propos de décisions importantes, de changements ou de projets futurs? |  | Sul posto di lavoro viene informato(a) con largo anticipo riguardo, ad esempio, decisioni importanti, cambiamenti o progetti per il futuro? |
| PRE2 | Do you receive all the information you need in order to do your work well? |  | Erhalten Sie alle Informationen, die Sie brauchen, um Ihre Arbeit gut zu erledigen? |  | Recevez-vous toutes les informations dont vous avez besoin pour bien faire votre travail? |  | Riceve tutte le informazioni di cui ha  bisogno per svolgere bene il Suo lavoro? |
|  |  |  |  |  |  |  |  |
| Rewards (REW) | | | | | | | |
| REW1 | Does the management at your workplace respect you? |  | Erfährt Ihre Arbeit Anerkennung und Wertschätzung durch das Management / die Führung? |  | Votre travail fait-il l'objet de reconnaissance et d'estime par le management / la direction? |  | Il Suo lavoro viene riconosciuto e apprezzato dal management e dalla direzione? |
|  |  |  |  |  |  |  |  |
| Role clarity (RCL) | | | | | | | |
| RCL1 | Does your work have clear objectives? |  | Gibt es klare Ziele für Ihre Arbeit? |  | Votre travail a-t-il des objectifs clairs? |  | Il Suo lavoro ha obiettivi chiari? |
| RCL2 | Do you know exactly which areas are your responsibility? |  | Wissen Sie genau, welche Dinge in Ihren Verantwortungsbereich fallen? |  | Savez-vous exactement quels sont les tâches sous votre responsabilité? |  | Sa esattamente quali sono le Sue aree di responsabilità? |
| RCL3 | Do you know exactly what is expected of you at work? |  | Wissen Sie genau, was von Ihnen bei der Arbeit erwartet wird? |  | Savez-vous exactement ce que l’on attend de vous dans votre travail? |  | Sa esattamente cosa ci si aspetta da Lei sul lavoro? |
|  |  |  |  |  |  |  |  |
| Role conflicts (RCF) | | | | | | | |
| RCF1 | Are contradictory demands placed on you at work? |  | Werden bei Ihrer Arbeit widersprüchliche Anforderungen gestellt? |  | Existe-t-il des exigences contradictoires dans votre travail? |  | Ci sono richieste contraddittorie che Le vengono fatte sul lavoro? |
| RCF2 | Do you sometimes have to do things, which ought to have been done in a different way? |  | Müssen Sie manchmal Dinge tun, die eigentlich auf andere Weise getan werden sollten? |  | Devez-vous parfois faire des choses qui devraient être accomplies de manière différente? |  | A volte deve fare cose che dovrebbero essere fatte in un modo diverso? |
| RCF3 | Do you sometimes have to do things, which seem to be unnecessary? |  | Müssen Sie manchmal Dinge tun, die Ihnen unnötig erscheinen? |  | Devez-vous parfois faire des choses qui vous semblent inutiles? |  | A volte deve fare cose che Le sembrano inutili? |
|  |  |  |  |  |  |  |  |
| Quality of leadership (QOL) | | | | | | | |
|  | To what extent would you say that your immediate superior… |  | Bitte schätzen Sie ein, in welchem Mass Ihr(e) unmittelbare(r) Vorgesetzte(r)… |  | Dans quelle mesure diriez-vous que vos supérieurs(es) hiérarchiques |  | Secondo lei, in quale misura i Suoi diretti superiori… |
| QOL1 | …makes sure that the individual member of staff has good development opportunities? |  | …für gute Entwicklungsmöglichkeiten der Mitarbeiter(innen) sorgt? |  | ...s’assurent que les membres  du personnel ont de bonnes  opportunités de développement? |  | ...si assicurano  che i singoli membri  del personale abbiano buone  opportunità di sviluppo? |
| QOL2 | … gives high priority to job satisfaction? |  | …der Arbeitszufriedenheit einen hohen Stellenwert beimisst? |  | ...donnent priorité à la satisfaction au travail? |  | ...danno massima priorità alla soddisfazione sul lavoro? |
| QOL3 | …is good at work planning? |  | …die Arbeit gut plant? |  | …planifient bien le travail? |  | …sono bravi a pianificare il lavoro? |
| QOL4 | … is good at solving conflicts? |  | …Konflikte gut löst? |  | …résolvent bien les conflits? |  | …sono bravi a risolvere i conflitti? |
|  |  | | | | |  |  |
| Social support at work (SOS) | | | | | | | |
| SOS1 | How often do you get help and support from your colleagues? |  | Wie oft erhalten Sie bei Bedarf Hilfe und Unterstützung von Ihren Kolleg(inn)en? |  | À quelle fréquence obtenez-vous au besoin, le soutien de vos collègues? |  | Quanto spesso riceve aiuto e supporto dai Suoi colleghi/e? |
| SOS2 | How often are your colleagues willing to listen to your work-related problems? |  | Wie oft sind Ihre Kolleg(inn)en bei Bedarf bereit, sich Ihre Arbeitsprobleme anzuhören? |  | À quelle fréquence vos collègues sont-ils disposés(es) à écouter vos problèmes au travail? |  | Quanto spesso i Suoi colleghi/e sono disponibili ad ascoltare i Suoi problemi legati al lavoro? |
| SOS3 | How often do you get help and support from your immediate superior? |  | Wie oft erhalten Sie bei Bedarf Hilfe und Unterstützung von Ihrem / Ihrer unmittelbaren Vorgesetzten? |  | À quelle fréquence obtenez-vous l’aide et le soutien de votre supérieur(e) direct(e)? |  | Quanto spesso riceve aiuto e supporto dal Suo (dalla Sua) diretto(a) superiore? |
| SOS4 | How often is your immediate superior willing to listen to your work-related problems? |  | Wie oft ist Ihr(e) unmittelbare(r) Vorgesetzte(r) bei Bedarf bereit, sich Ihre Arbeitsprobleme anzuhören? |  | À quelle fréquence votre supérieur(e) est-il(elle) disposé(e) à écouter vos problèmes liés au travail? |  | Quanto spesso il Suo (la Sua) diretto(a) superiore è disponibile ad ascoltare i Suoi problemi legati al lavoro? |
|  |  |  |  |  |  |  |  |
| Feedback (FEB) | | | | | | | |
| FEB1 | How often does your superior talk with you about how well you carry out your work? |  | Wie oft spricht Ihr(e) Vorgesetzte(r) mit Ihnen über die Qualität Ihrer Arbeit? |  | À quelle fréquence votre supérieur(e) s'entretient-il(elle) avec vous de la qualité de votre travail? |  | Quanto spesso parla con il Suo (la Sua) diretto(a) superiore della qualità del Suo lavoro? |
| FEB2 | How often do your colleagues talk with you about how well you carry out your work? |  | Wie oft sprechen Ihre Kolleg(inn)en mit Ihnen über die Qualität Ihrer Arbeit? |  | À quelle fréquence vos collègues s'entretiennent-ils(elles) avec vous de la qualité de votre travail? |  | Quanto spesso parla con i Suoi colleghi/e della qualità del Suo lavoro? |
|  |  |  |  |  |  |  |  |
| Social relations at work (SRW) | | | | | | | |
| SRW1 | Is it possible for you to talk to your colleagues while you are working? |  | Können Sie sich mit Kolleg(inn)en unterhalten, während Sie arbeiten? |  | Vous est-il possible de parler avec vos collègues pendant que vous travaillez? |  | Le è possibile parlare con i Suoi colleghi(e) mentre è al lavoro? |
|  |  | | | | |  |  |
| Social community (SCW) | | | | | | | |
| SCW1 | Is there a good atmosphere between you and your colleagues? |  | Ist die Atmosphäre zwischen Ihnen und Ihren Arbeitskollegen(inn)en gut? |  | L’ambiance entre vous et vos collègues est-elle bonne? |  | C’è una buona atmosfera tra Lei e i Suoi colleghi(e)? |
| SCW2 | Is there good co-operation between the colleagues at work? |  | Ist die Zusammenarbeit zwischen den Arbeitskollegen(inn)en gut? |  | La collaboration entre les collègues de travail est-elle bonne? |  | C’è buona cooperazione tra i Suoi colleghi(e) sul posto lavoro? |
|  |  |  |  |  |  |  |  |
| Unfair behaviour / mobbing (UNB) | | | | | | | |
| UNB | How often do you feel unfairly threatened, criticized, harassed or exposed by your colleagues and supervisors? |  | Wie oft fühlen Sie sich durch Kollegen(inn)en oder Vorgesetzte zu Unrecht kritisiert, schikaniert oder vor anderen blossgestellt? |  | À quelle fréquence vous sentez-vous injustement critiqué(e), harcelé(e) ou humilié(e) devant les autres par vos collègues ou votre supérieur(e) hiérarchique ? |  | Quanto spesso si sente ingiustamente criticato(a), oggetto di soprusi o additato/a dai Suoi colleghi(e) e dal Suo(dalla Sua) superiore? |
|  |  |  |  |  |  |  |  |
| **Person-work interface** | | | | | | | |
| Job insecurity (JIS) | | | | | | | |
| JIS1 | Are you worried about becoming unemployed? |  | Machen Sie sich Sorgen, dass Sie arbeitslos werden? |  | Êtes-vous inquiet(ète) de vous retrouver au chômage? |  | Si preoccupa di rimanere disoccupato(a)? |
| JIS2 | Are you worried about new technology making you redundant? |  | Machen Sie sich Sorgen, dass neue Technologien Sie überflüssig machen? |  | Êtes-vous inquiet(ète) qu'une nouvelle technologie vous rende superflu(e)? |  | Si preoccupa che le nuove tecnologie La rendano superflua/o? |
| JIS3 | Are you worried about it being difficult for you to find another job if you became unemployed? |  | Machen Sie sich Sorgen, dass es schwierig für Sie wäre, eine neue Arbeit zu finden, wen Sie arbeitslos würden? |  | Êtes-vous inquiet(ète)… …que l'on puisse vous transférer à un autre poste contre votre volonté? |  | Si preoccupa… …di essere trasferito(a) ad un altro posto di lavoro contro la Sua volontà? |
| JIS4 | Are you worried about being transferred to another job against your will? |  | Machen Sie sich Sorgen, dass man Sie gegen Ihren Willen auf eine andere Arbeitsstelle versetzen könnte? |  | Êtes-vous inquiet(ète)… …que l'on puisse vous transférer à un autre poste contre votre volonté? |  | Si preoccupa…di essere trasferito(a) ad un altro posto di lavoro contro la Sua volontà? |
|  |  |  |  |  |  |  |  |
| Insecurity of the working environment (IWE) | | | | | | | |
| IWE1 | Are you worried about… … the timetable being changed (shift,  weekdays, time to enter and leave, ...)  against your will? |  | Machen Sie sich Sorgen… …Ihre Arbeitszeiten gegen Ihren Willen verändert werden  (z.B. Arbeitstage, Schichtpläne, Arbeitsbeginn- und ende?) |  | Êtes-vous inquiet(ète)…que vos horaires de travail soient modifiés contre votre volonté (p. ex. jours de travail, horaires, début et fin du travail)? |  | Si preoccup... che i Suoi orari di lavoro possano essere modificati contro la Sua volontà? (per esempio i giorni di lavoro, i turni, l'orario di inizio/fine turno?) |
| IWE2 | Are you worried about… a decrease in your salary |  | Machen Sie sich Sorgen, dass Ihr Lohn / Gehalt verringert werden könnte? |  | ...que votre salaire ou votre rémunération puisse être diminué? |  | ...che il Suo stipendio possa essere ridotto? |
|  |  |  |  |  |  |  |  |
| **Home-work interface** | | | | | | | |
| Work-private life conflict (WPC) | | | | | | | |
| WPC1 | The demands of my work interfere with my home and family life. |  | Die Anforderungen meiner Arbeit stören mein Privat-/ und Familienleben. |  | Les exigences de mon travail interfèrent avec ma vie privée et familiale. |  | Le esigenze del mio lavoro interferiscono con la mia vita domestica e familiare. |
| WPC2 | The amount of time my job takes up makes it difficult to fulfil my family responsibilities |  | Der Zeitaufwand meiner Arbeit macht es schwierig für mich, meinen Pflichten in der Familie oder im Privatleben nachzukommen. |  | Il m’est difficile de remplir mes responsabilités familiales en raison du temps consacré à mon travail. |  | Il tempo che mi prende il lavoro rende difficile l’adempimento delle mie responsabilità familiari. |
| WPC3 | Do you feel that your work drains so much of your energy that it has a negative effect on your private life? |  | Meine Arbeit beansprucht so viel Energie, dass sich dies negativ auf mein Privatleben auswirkt. |  | Mon travail demande tant d'énergie qu'il impacte de manière négative sur ma vie privée. |  | Il mio lavoro richiede così tante energie, che interferisce in maniera negativa sulla mia vita privata. |
| WPC4 | Do you feel that your work takes so much of your time that it has a negative effect on your private life? |  | Meine Arbeit nimmt so viel Zeit in Anspruch, dass sich dies negativ auf mein Privatleben auswirkt. |  | Mon travail demande tant de temps qu'il impacte de manière négative sur ma vie privée. |  | Il mio lavoro richiede così tanto tempo che interferisce in maniera negativa sulla mia vita privata |
| WPC5 | It happens that I should be at home and at work at the same time. |  | Es kommt vor, dass ich zur gleichen Zeit zu Hause und bei der Arbeit sein sollte. |  | Il arrive que je doive être en même temps à la maison et à mon travail. |  | Succede che dovrei essere... |
|  |  |  |  |  |  |  |  |
| Problems with demarcation (DEM) | | | | | | | |
| DEM1 | I take care of work-related tasks outside of my working time as well. |  | Ich erledige berufliche Dinge auch ausserhalb meiner Arbeitszeit. |  | J'effectue également des tâches professionnelles en dehors de mon temps de travail. |  | Svolgo incombenze professionali anche fuori dal mio orario di lavoro. |
| DEM2 | I’m available in my free time for people with whom I deal professionally |  | In meiner Freizeit bin ich für Personen, mit denen ich beruflich zu tun habe, erreichbar. |  | Durant mon temps libre, je suis joignable pour les personnes avec lesquelles j'ai à faire professionnellement. |  | Nel mio tempo libero sono raggiungibile per persone con le quali ho a che fare per lavoro. |
|  |  |  |  |  |  |  |  |
| **Stress symptoms and long-term consequences** | | | | | | | |
| Behavioural stress symptoms (BSS) | | | | | | | |
|  | Please review the statements below and check which answer best applies to you for the last four weeks. (Please indicate one statement per line) |  | Bitte betrachten Sie die nachfolgenden Aussagen und kreuzen Sie jeweils an, welche Antwort für die letzten vier Wochen am besten auf Sie zutrifft. (Bitte je eine Angabe pro Zeile) |  | Veuillez lire les affirmations ci-dessous et cocher la réponse qui s’applique le mieux à votre cas au cours des quatre dernières semaines. (Veuillez fournir une réponse à chaque ligne.) |  | Per favore legga le seguenti affermazioni e segni il valore che più rispecchia le ultime quattro settimane. (Per favore dare una risposta per ciascuna/ogni riga) |
| BSS1 | I have not been able to stand dealing with other people. |  | Ich hatte nicht die Energie, mich mit anderen Leuten zu beschäftigen. |  | Je n'avais pas l'énergie de m'occuper d'autres personnes. |  | Non ho avuto le energie per dedicarmi ad altre persone. |
| BSS2 | I have not had the time to relax or enjoy myself. |  | Ich hatte nicht die Zeit, mich zu entspannen oder mich zu vergnügen. |  | Je n'avais jamais le temps de me reposer ou de me divertir. |  | Non ho avuto il tempo per rilassarmi o divertirmi. |
| BSS3 | I have been a bit touchy |  | Ich war leichter aus der Bahn zu werfen. |  | J’étais plus susceptible. |  | Mi sentivo emotivamente instabile. |
| BSS4 | I have lacked initiative. |  | Ich war antriebslos. |  | Je manquais d’entrain. |  | Ero senza energia. |
|  |  |  |  |  |  |  |  |
| Cognitive stress symptoms (CSS) | | | | | | | |
|  | How much of the time during the past 4 weeks have you |  | Wie oft hatten Sie in den vergangenen vier Wochen… (Bitte je eine Angabe pro Zeile) |  | Combien de fois, au cours des 4 dernières semaines, avez-vous... |  | Quante volte nelle ultime 4 settimane ha: |
| CSS1 | had problems concentrating? |  | …Konzentrationsprobleme? |  | ...eu des problèmes de  concentration? |  | ...avuto problemi a concentrarsi? |
| CSS2 | had difficulty in taking decisions? |  | …Schwierigkeiten, Entscheidungen zu treffen? |  | ...eu des difficultés à prendre des décisions? |  | ...avuto difficoltà a prendere decisioni? |
| CSS3 | had difficulty with remembering? |  | …Schwierigkeiten, sich zu erinnern? |  | ...eu des difficultés de mémoire? |  | ...avuto difficoltà a ricordare? |
| CSS4 | found it difficult to think clearly? |  | …Schwierigkeiten, klar zu denken? |  | …trouvé difficile de penser clairement? |  | …avuto difficoltà a pensare con chiarezza? |
|  |  |  |  |  |  |  |  |
| Job satisfaction (JSA) | | | | | | | |
|  | Regarding your work in general. How pleased are you with… |  | Wenn Sie Ihre Arbeitssituation insgesamt betrachten, wie zufrieden sind Sie mit… |  | En considérant votre situation de travail dans son ensemble, dans quelle mesure êtes-vous satisfait(e)... |  | Riguardo al Suo lavoro in generale. Quanto è soddisfatto(a) di… |
| JSA1 | ...your work prospects? |  | …Ihren Berufsperspektiven? |  | ...de vos perspectives professionnelles |  | ...le Sue prospettive di lavoro? |
| JSA2 | …the people you work with? |  | …den Leuten, mit denen Sie arbeiten? |  | ...des conditions physiques de travail? |  | ...le condizioni fisiche di lavoro? |
| JSA3 | ...the physical working conditions? |  | …den körperlichen Arbeitsbedingungen? |  | ...des conditions physiques de travail? |  | ...le condizioni fisiche di lavoro? |
| JSA4 | ...the way your group is run? |  | …der Art und Weise, wie Ihre Abteilung geführt wird? |  | ...de la manière dont votre service fonctionne? |  | ...il modo in cui è gestito il Suo settore? |
| JSA5 | ...the way your abilities are used? |  | …der Art und Weise, wie Ihre Fähigkeiten genutzt werden? |  | ...de la manière dont vos capacités sont utilisées? |  | ...il modo in cui sono impiegate le Sue capacità? |
| JSA6 | ...your job as a whole, everything taken into consideration? |  | …Ihrer Arbeit insgesamt, unter Berücksichtigung aller Umstände? |  | ...de votre travail dans son ensemble, en prenant toutes les circonstances en considération? |  | ...il suo lavoro nel complesso, prendendo in considerazione tutto? |
|  |  |  |  |  |  |  |  |
| Intention to leave the organisation (ILO) | | | | | | | |
| ILO1 | In the past 12 months, how often have you thought about… … changing your job? |  | Wie oft haben Sie im Laufe der letzten 12 Monate daran gedacht, Ihre Arbeitsstelle zu wechseln? |  | Combien de fois, au cours des 12 derniers mois, avez-vous pensé à quitter votre place de travail ? |  | Nel corso degli ultimi 12 mesi quante volte ha pensato di cambiare posto di lavoro? |
|  |  |  |  |  |  |  |  |
| Intention to leave the profession (ILP) | | | | | | | |
| ILP1 | In the past 12 months, how often have you thought about… … giving up your profession? |  | Wie oft haben Sie im Laufe der letzten 12 Monate daran gedacht, Ihren Beruf aufzugeben? |  | Combien de fois, au cours des 12 derniers mois, avez-vous pensé à quitter votre métier? |  | Quante volte, nel corso dell'anno passato, ha pensato di abbandonare la sua professione? |
|  |  |  |  |  |  |  |  |
| Burnout-symptoms (BUS) | | | | | | | |
| BUS1 | How often do you feel physically exhausted? |  | Wie häufig sind Sie körperlich erschöpft? |  | À quelle fréquence vous sentez-vous physiquement fatigué(e)? |  | Con che frequenza si sente fisicamente esaurito(a)? |
| BUS2 | How often do you feel emotionally exhausted? |  | Wie häufig sind Sie emotional erschöpft? |  | À quelle fréquence vous sentez-vous émotionnellement fatigué(e)? |  | Con che frequenza si sente emotivamente esaurito(a)? |
| BUS3 | How often did you feel worn out? |  | Wie häufig fühlen Sie sich ausgelaugt? |  | À quelle fréquence vous êtes-vous senti(e) épuisé(e)? |  | Con che frequenza si sente logorato(a)? |

Kristensen (2000), Nübling, Stößel, Hasselhorn, Michaelis, and Hofmann (2005), Nübling et al. (2017)
