# Supplementary material for: Assessing the psychosocial work environment in the health care setting: translation and psychometric testing of the French and Italian Copenhagen Psychosocial Questionnaires (COPSOQ) in a large sample of health professionals in Switzerland
Source: BMC Health Serv Res. 2022 May 6;22:608. doi: 10.1186/s12913-022-07924-4 (PMC9074249; doi:10.1186/s12913-022-07924-4)
Supplement: Supplementary file 2 — Additional file 2: Supplement B. Participants and setting characteristics stratified by language (German, French, Italian). [file 12913_2022_7924_MOESM2_ESM.docx]

| **Supplement B:** Participants and setting characteristics stratified by language (German, French, Italian) | | | | |
| --- | --- | --- | --- | --- |
|  |  |  |  |  |
|  | **German** | **French** | **Italian** | **Total** |
|  | N (%) | N (%) | N (%) | N (%) |
|  |  |  |  |  |
| **Total participants** | 10738 (84.2) | 1788 (14.0) | 228 (1.8) | 12754 |
|  |  |  |  |  |
| **Sex** |  |  |  |  |
| Female | 8725 (81.3) | 1444 (81.0) | 156 (68.4) | 10325 (81) |
| Missing | 175 (1.6) | 21 (1.2) | 2 (0.9) | 198 (1.6) |
|  |  |  |  |  |
| Age yrs. (mean, SD) | 41.52 (12.6) | 41.23 (11.6) | 41.55 (11.2) | 41.48 (12.5) |
|  |  |  |  |  |
| **Profession** |  |  |  |  |
| Nurses | 6521 (60.7) | 956 (53.5) | 116 (50.9) | 7593 (59.5) |
| Midwifery | 102 (0.9) | 11 (0.6) | 0 (0) | 113 (0.9) |
| Medical-technical professions | 304 (2.8) | 81 (4.5) | 0 (0) | 385 (3) |
| Medical-therapeutic professions | 816 (7.6) | 125 (7) | 16 (7) | 957 (7.5) |
| Physicians | 694 (6.5) | 89 (5) | 31 (13.6) | 814 (6.4) |
| Administration/Research | 215 (2) | 47 (2.6) | 10 (4.4) | 272 (2.1) |
| Social Service | 224 (2.1) | 28 (1.6) | 1 (0.4) | 253 (2) |
| Domestic help | 150 (1.4) | 34 (1.9) | 54 (23.7) | 184 (1.4) |
| Other | 1712 (15.9) | 417 (23.3) | 0 (0) | 2183 (17.1) |
|  |  |  |  |  |
| **Setting** |  |  |  |  |
| Acute care hospital | 4500 (42.0) | 957 (53.5) | 0 (0) | 5457 (42.8) |
| Rehabilitation hospital | 45 (0.4) | 52 (2.9) | 0 (0) | 97 (0.8) |
| Psychiatry hospital | 2943 (27.4) | 136 (7.6) | 122 (53.5) | 3201 (25.1) |
| Nursing home | 1905 (17.7) | 490 (27.4) | 71 (31.1) | 2466 (19.3) |
| Home-care organisation | 1345 (12.5) | 153 (8.6) | 35 (15.4) | 1533 (12.0) |
|  |  |  |  |  |
